# Protein Biomaterials with Muscle-like Water-Driven Actuation

**DOI:** 10.1021/acsami.5c19991

**Published:** 2026-01-03

**Authors:** Sanam Bista, Ionel Popa

**Affiliations:** Department of Physics and Astronomy, 14751 University of Wisconsin-Milwaukee, 3135 N. Maryland Ave, Milwaukee, Wisconsin 53211, United States

**Keywords:** protein-based biomaterials, solvent-responsive actuation, shape-memory, amyloid fibrils, solvent-exchange motor, programmable biomaterials, hydrogel actuators, Marangoni flow

## Abstract

Unlike muscles, man-made shape-morphing biomaterials take much longer times to perform their actuation. Here we report a novel class of protein-based actuators that mimic muscle contraction through ethanol-induced fibril formation in bovine serum albumin (BSA) hydrogels, enabling reversible shape changes and fast, water-driven motion. These structural changes result in mechanical stiffening, enabling programmable and reversible shape changes, which take place over minutes to hours. At intermediate ethanol concentrations (40–80%), fibril formation dominates and contributes to shape retention, while at high ethanol concentrations (80–99%), aggregation outpaces fibrillation, allowing full recovery of the original shape upon rehydration. Furthermore, upon reinsertion into water, ethanol retention triggers stochastic pulsating motion in cylindrical samples and spins on the a protein-based propeller motor (rotational speeds up to 471 deg·s^–1^), a process driven by a surface tension gradient. These findings address the challenge of achieving rapid, reversible motion in biomaterials, resembling that of muscles, with promising applications in smart biomaterials, microactuators, and bioresponsive systems.

## Graphical abstract

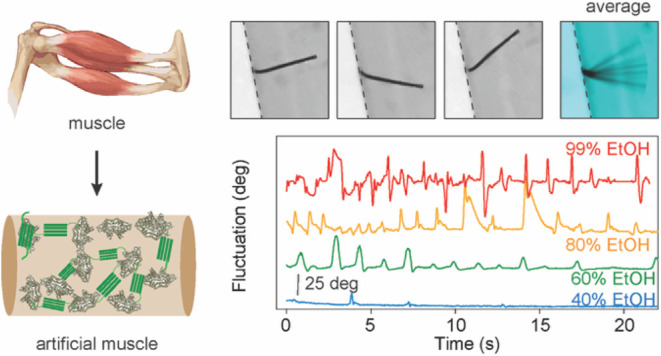

## 1 Introduction

Biological-like motion can enable biomaterials to mimic the dynamic and responsive movements found in living organisms. In nature, motion is related to the structural properties of tissues, such as muscles, which consume energy to turn on molecular motors such as myosin and rely on conformational changes such as the unfolding-refolding transitions seen in the muscle spring protein titin, to optimize energy expenditure (Figure 1A).^1^ Muscles can develop kN forces through their structured architecture. During contraction, at the molecular level, the polyproteins inside muscles unfold some of their domains or change their conformation, and this process is driven by the presence or absence of calcium ions.^1^ In biomaterials, biological motion has been achieved through double-network designs or biological mimics. The double-network design uses the primary network, which is typically static, for maintaining structural integrity, while the secondary network, which is typically dynamic, can be employed to produce programmed shapes and drive motion.^2–5^ To achieve programmed shapes, a significant change in the overall stiffness of the material is required, and this change needs to be reversible to produce the transition back from the programmed to the original shape.^5^ Several double-network designs have incorporated protein components such as elastin, collagen, or other peptides inside polymeric matrices.^6–10^ In one unique approach, the primary network made from folded proteins was shown to drive the motion through solvent-induced unfolding, while the secondary polymeric network (unstructured by nature) maintained the structural integrity during perturbation.^11^ However, the large majority of applications produce actuation on the minute time scale, much slower than human muscles.

**Figure 1:**
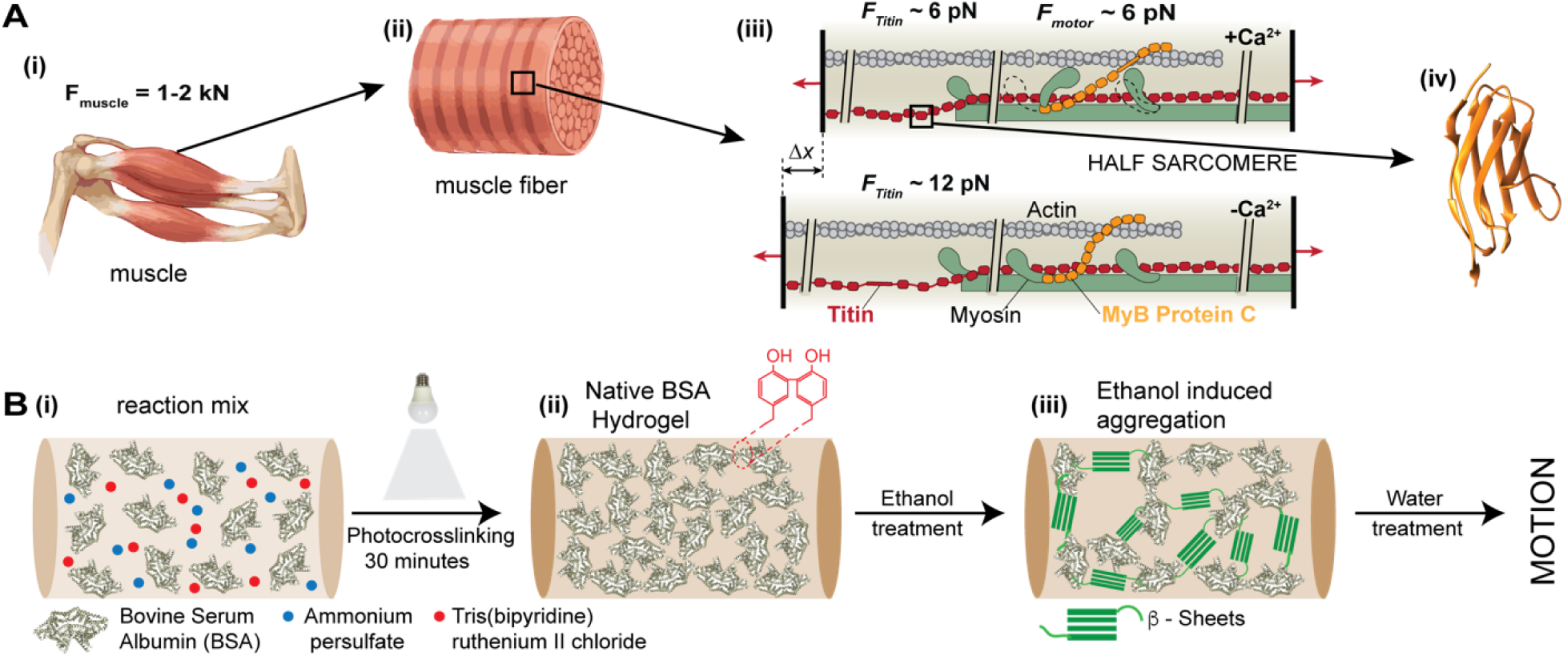
Schematics of the approach adopted in this study, inspired from the architecture of muscles. **A)** Biological architecture of striated muscles: **(i)** rendering of a muscle, capable of applying kN force; **(ii)** mesoscopic-level organization of muscle fibers into sarcomeres; (**iii**) diagram showing a half-sarcomere with actin filaments on one side, myosin motors on the opposite side, linked longitudinally by titin and transversally by myosin-binding protein C; (**iv**) ribbon structure of the titin I91 domain (PDB: 1tit); **B) (i)** to **(ii)**: Synthesis approach using pure globular proteins alongside an oxidant and catalyst to produce biomaterials through light-induced cross-linking; **(ii)** to **(iii)** in the presence of ethanol, which is a poor solvent for proteins, the internal structure of the protein biomaterial changes, influencing its biomechanical properties; inset in **(ii)** shows two tyrosine amino acids cross-linked in their ortho orientation; inset in **(iii)** shows a β-sheet structure induced by immersion in alcohol.

Apart from the double-network design often employed to engineer materials that display biological motion, another venue is represented by reversible or irreversible changes in the internal structure of the molecules forming the biomaterials, driven by their interaction with solvent molecules. Such an example was shown for folded proteins, where the presence of divalent ions in high concentrations (several molar) could make negatively charged proteins condense and stiffen.^12–14^ Similarly, using the effect of a poor-solvent, it was shown that biomaterials made from unstructured silk polypeptides can change their random-coil internal structure to form β-sheets and fibrillar structures in the presence of methanol.^15^ The formation of fibrillar structures is a complex process, which typically proceeds through some protein denaturation or partial unfolding, followed by nucleation through soluble oligomeric “seeds”, that drive the formation of protofibrils and mature fibrils; these protofibrils associate laterally and rearrange to form highly ordered, insoluble fibrillar structures, often with a characteristic cross-β architecture.^16^ The ability to scale up the formation of nonpathogenic protein fibrils is essential for developing novel biomaterials, necessitating new, controllable methods for producing these fibrillar structures at scale.^17,18^

Ethanol was shown to change the tertiary structure of proteins such as bovine serum albumin (BSA), interacting favorably with hydrophobic residues and promoting denaturation and soluble oligomers.^19,20^ The initial binding of ethanol to BSA takes place at high-affinity sites, located in the hydrophobic pockets of the protein, with the methyl group facing the protein core and the hydroxyl group facing the solvent environment.^19,21^ Upon further increase in ethanol concentration, ethanol binds to multiple nonspecific low-affinity sites, triggering the denaturation of BSA.^21^ This denaturation is accompanied by a decrease in solubility^20^ and can lead to the formation of insoluble fibrillar structures. This hypothesis is also supported by other studies, where concentrated solutions of BSA in the presence of other destabilizing agents, such as pH, temperature, or cosolvent, were shown to trigger the formation of fibers.^22–26^ While alcohol-induced fibrillation of BSA in solution has been reported previously,^22^ leading to β-sheet-rich aggregates under thermal or solvent stress, our work extends this to covalently cross-linked hydrogels, enabling tunable mechanical stiffening, programmable shape morphing, and rapid (second-timescale) solvent-exchange actuation not observed in prior solution-based studies.

Here we explore the possibility of programming protein-based biomaterials using the interaction of the network-forming molecules with alcoholic solvents that can drive fiber formation inside biomaterials. More specifically, we investigated the effect of ethanol on the physical properties of protein-based biomaterials synthesized from bovine serum albumin (BSA). We find that ethanol has a surprisingly stiffening effect on the BSA-based biomaterials, which is largest in the 60–80% ethanol concentration range. We correlate this effect with fibril formation inside the biomaterial. Using the interaction between BSA and ethanol, we demonstrate shape morphing by programming BSA-based biomaterials driven by solvent exchange. Finally, we report on a fast-fluctuating motion seen upon immersion of BSA-based biomaterials from ethanol into water, which we correlate to a solvent-induced surface tension gradient. Using reversible structural changes and solvent flow to produce biologically-like motion in protein-based biomaterials is an important step toward developing muscle mimics and environmentally adaptive soft actuators.

## 2 Materials and Methods

### 2.1 Materials

Bovine Serum Albumin (BSA) was purchased from Rocky Mountain Biologicals. Sodium chloride was purchased from Thermo Fisher Scientific. 1-Anilinonaphthalene-8-Sulfonic Acid (1,8-ANS) was purchased from Cayman Chemical Company. All other chemicals were purchased from Sigma-Aldrich. Saline buffer (20 mM Tris and 150 mM NaCl with a pH 7.4) was used as the buffer.

### 2.2 Synthesis of BSA-Based Biomaterials

In this study, we synthesized BSA protein biomaterials, following the same procedure as in our previous studies.^13,27^ Briefly, BSA powder was dissolved in saline buffer (20 mM Tris and 150 mM NaCl) with a pH of 7.4 to a concentration of 2 mM. For the oxidant and initiator solutions, ammonium persulfate (APS) (1 M) and Tris­(bipyridine)­ruthenium­(II) chloride ([Ru­(bpy)_3_]^2+^) (6.67 mM) salt were prepared by dissolving the powders in saline buffer. Before biomaterial synthesis, the protein solution was mixed with APS and [Ru­(bpy)_3_]^2+^ in a volume ratio of 15:1:1, vortexed to homogenize, and centrifuged to remove bubbles. This solution mix was then either injected in a plastic tube with a 280 μm inner radius to obtain a cylindrical gel or placed in a mold made from dragonskin, to produce the disk-shaped, flower-shaped, or six-arm propeller samples. Before adding the reaction mix, the molds were treated with Sigmacote for 5 min, to minimize adhesion to the walls. The polymerization was done for 30 min under a 100 W mercury lamp placed 30 cm away and having a filter to block UV radiation (400 nm long-pass). The irradiance at 660 nm, where [Ru­(bpy)_3_]^2+^ has maximum absorption, was 1.6 mW/cm^2^. Following polymerization, the newly formed protein biomaterials were taken out from the molds and submerged in a saline buffer solution.

### 2.3 Mechanical Characterization

The 2 mM BSA biomaterials were first equilibrated with saline buffer at room temperature for over 30 min until use. Their viscoelastic response was characterized using force clamp rheometry.^13,27^ Briefly, a cylindrical biomaterial of 3–5 mm in length was attached between two hooks connected to a voice coil and a force sensor, respectively, using surgical sutures and immersed in a hexagonal weighing boat filled with saline buffer. Following the first stress–strain application, the same sample was immersed in double-distilled (DDI) water for 30 min, and its mechanical response was measured once more. Finally, the sample was incubated into a buffer containing a specific concentration of ethanol (20–60% v/v) for an additional 30 min, and its response was measured once more. The mechanical response of both the native and ethanol treated BSA-based samples was tested using a force-ramp protocol, where the stress was linearly increased and decreased over time at a controlled change rate of 40 Pa/s. An analog proportional-integral-differential (PID) system was used to continuously adjust the voice coil position to match the desired stress. The Young’s modulus was calculated using a linear fit of the stress vs strain, on the force-loading part of the curve, in the linear region. The energy dissipation was estimated from the hysteresis between the stress increase and the decrease.

### 2.4 Shape Programming and Morphing

A flower-shaped BSA-based material, prepared as previously described, was first equilibrated with saline buffers to preserve its initial flower shape, followed by incubation in distilled deionized (DDI) water for 30 min to remove the salt. To program and change the structure of the flower-shaped biomaterial into a ring shape, the sample was mounted on a 5 mL tube, which was then immersed in a buffer containing ethanol for 30 min. Subsequently, the biomaterials were removed from the tube and placed in the same concentration of ethanol for an additional 30 min. Then the materials were transferred back to DDI water for 30 min, followed by saline buffer. The changes in shape were monitored by using time-lapse recordings.

### 2.5 Observations of Programmed V-Shaped Recovery

BSA-based materials were synthesized inside the PTFE tubes, extruded in saline buffer, transferred into DDI water for 30 min, and utilized to program a V-shape by using a 3D-printed mold, followed by incubating 30 min in different concentrations of ethanol. Thereafter, the biomaterials were removed from the mold and incubated in the same concentration of ethanol for 30 min. Following this step, the V-shaped materials were placed in DDI water for 30 min and then transferred back to the saline buffer for another 30 min. The samples were observed and photographed every 5 min, and the angle was quantified from the position of the two arms forming the letter “V”. Data was analyzed using a custom software written in Igor Pro (Wavemetrics).

### 2.6 Scanning Electron Microscopy (SEM) Imaging

SEM imaging was conducted to observe the topology and the internal structure of both native BSA (2 mM) protein-based biomaterial and those treated with different concentrations of ethanol using a HITACHI S-4800 instrument. The cylindrical BSA-based biomaterials were synthesized as described previously, equilibrated in saline buffer, and transferred to the DDI water incubator for 30 min. For the preparation of native BSA-biomaterial samples, the BSA-biomaterial was incubated in DDI water for 30 min, followed by another 30 min step in the desired ethanol concentration, then flash frozen with liquid nitrogen and lyophilized overnight. The samples were then fractured to expose their interior cross-sectional area and attached on top of aluminum stubs using double-sided carbon tape. A 3 nm iridium coating was then applied using a sputter coater (Emitech K575 K).

### 2.7 Fluorescence Measurement of BSA (2 mM) Aggregation and Fibrillation inside Biomaterials

To investigate ethanol-induced aggregation and fibrillation of bovine serum albumin (BSA) within biomaterials, we prepared a disk-shaped BSA-based biomaterial using a 3D-printed Dragon skin mold with a diameter of 7.80 mm and a height of approximately 3 mm. The fluorescent dyes 8-Anilinonaphthalene-1-sulfonic acid (ANS) and Thioflavin T (ThT) were employed to monitor amorphous and amyloid-like aggregation under varying ethanol concentrations. For ANS experiments, ANS (1 μM) (prepared by diluting a 10 mM stock solution) was freshly prepared in various concentrations of ethanol ranging from 0% to 99% (v/v) and applied to each BSA protein biomaterial. Fluorescence images were captured at different time points by using a Syngene G:Box imaging system. Similarly, amyloid formation was monitored by applying freshly prepared ThT (1 μM) in ethanol (0–99%) to individual disk-shaped BSA-based biomaterial, and fluorescence images were acquired over time using the same system with appropriate excitation and emission filters. Fluorescence intensities were quantified by performing a line profile analysis (Igor Pro, Wavemetrics). For both ANS and ThT measurements, fluorescence intensities were normalized to the corresponding intensity values at 0 min exposure to the fluorescent dyes in ethanol of each biomaterial to account for baseline variation.

### 2.8 Protein Propellers

Six-arm propeller-shaped BSA biomaterials were synthesized using a custom-designed six-arm Dragon skin mold to explore the ethanol–water solvent exchange-induced motion. The positive shape was 3D printed in PLA (“.stl” file shared as part of Supporting Information), and used to fabricate the Dragon skin mold. A mixture of BSA protein (2 mM), APS, and [Ru­(bpy)_3_]^2+^ was carefully loaded into the mold without introducing air bubbles. The mold filled with the reaction mix was then covered with a glass coverslip on top and exposed under the mercury lamp for 30 min at room temperature to initiate photo-cross-linking. Following gelation, six-arm propeller-shaped BSA-based biomaterials were taken out from the molds and transferred into saline buffer, followed by 30 min of incubation in double-distilled (DDI) water. Subsequently, the BSA-biomaterial was submerged in one of the (40–99%) ethanol solutions for at least 30 min. To trigger solvent-exchange-induced motion, the six-arm BSA-based biomaterials were transferred from ethanol back into DDI water, where real-time spinning motion was observed and recorded. The resulting videos were analyzed using Igor Pro (Wavemetrics) to quantify the rotation angles of the BSA-based biomaterials incubated in 60–99% ethanol.

## 3 Results

### 3.1 Overview of Approach

As alcohols can have a significant effect on the structure and solubility of proteins, we hypothesized that BSA-made biomaterials could show a significant change in internal structure when exposed to ethanol-containing solvents. To check this hypothesis, we pursued the approach shown in Figure 1B. First, we synthesized protein-based biomaterials using a mixture of 2 mM BSA, an oxidizing agent (Ammonium persulfate - APS) and a catalyst ([Ru­(bpy)_3_]^2+^), which, under light activation, leads to the formation of a covalently bound network (Figure 1 (i) to (ii)). Cross-linking connections form at exposed tyrosine sites through carbon–carbon bonds.^28^ With over 80% solvent content,^11,29^ these materials function as hydrogels; since the constituent BSA molecules are solvent-exposed, any solvent exchange will have immediate effects throughout the material. Hence, in the following step, when exposed to a solvent containing ethanol, we expect that fibrillar structures will form inside the BSA-biomaterial, leading to significant changes in the stiffness (Figure 1 (ii) to (iii)). These changes in stiffness may then be used to reshape these biomaterials into a “programmed” shape. Furthermore, if these changes do not trigger significant breaking of the network backbone bonds, they could be reversed upon removal of ethanol, to transition back from the programmed to the original shape.

To understand the structural changes induced by ethanol, we started by characterizing its topography with scanning electron microscopy (SEM). As shown in Figure 2A, we imaged sectioned BSA-based biomaterials exposed to various ethanol concentrations. We noticed similar overall mesoscopic structures but also the appearance of fibers for the samples exposed to over 40% ethanol (Figure 2A). These fibrils had a diameter of ∼24 nm (Figure S1). Similar fibrils with 20–35 nm were observed by heating concentrated BSA solutions, when denatured in ionic liquids.^26^ Another interesting finding is given by the 99% ethanol-exposed sample. In this case, there are only pockets of fibers arranged between smooth zones (Figure 2A top right). Such smooth surfaces are seen also for 80% ethanol but over a much smaller area. These images suggest that additional morphological changes take place alongside fibril formation, most likely coming from insoluble amorphous assemblies.

**Figure 2:**
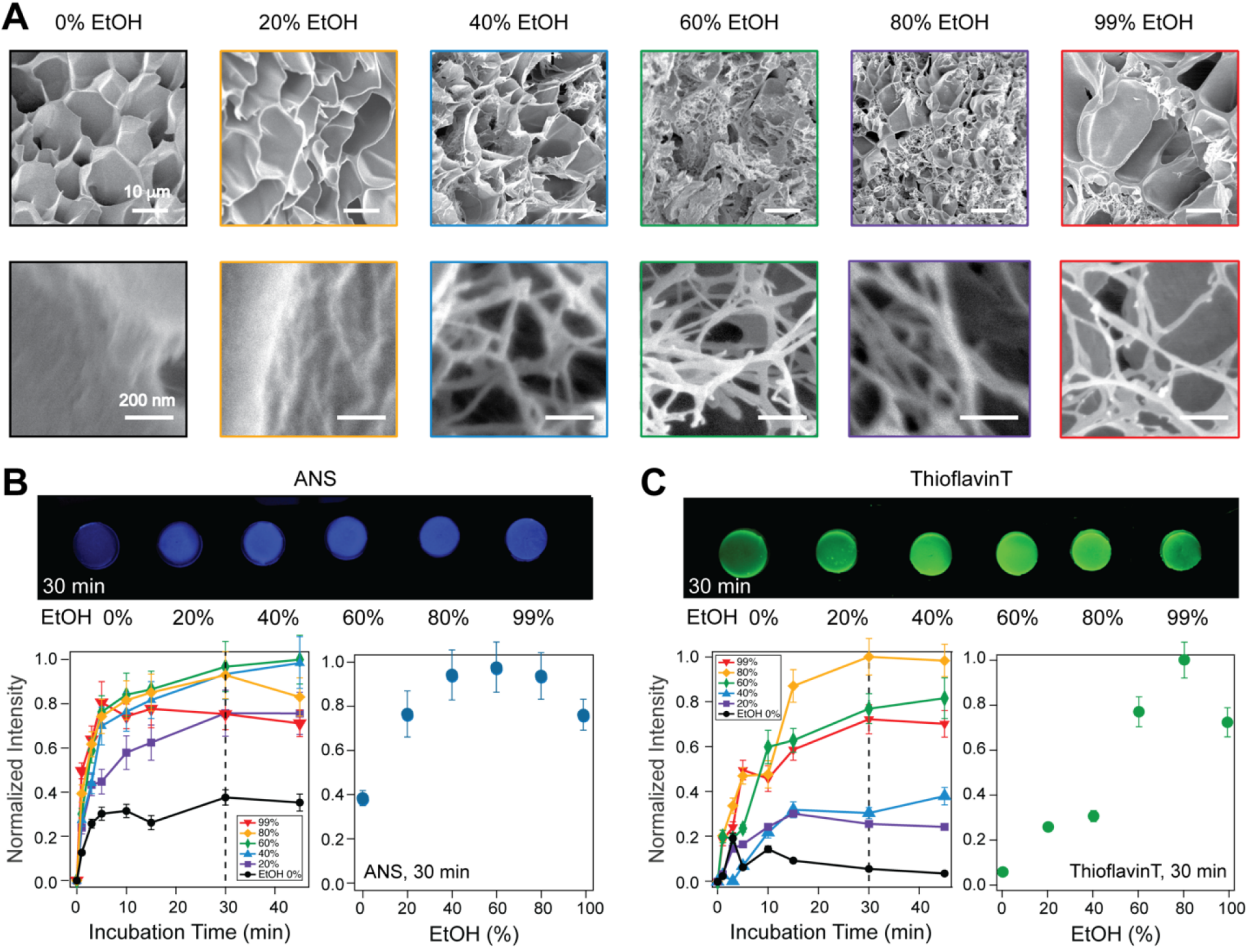
Structural changes induced by treatment of protein-based materials with ethanol. **A)** Scanning electron microscopy (SEM) images of BSA-based biomaterials showing the appearance of fibrous structures with increased ethanol concentration, after a 30 min exposure incubated overnight; scale bar: 10 μm; **B)** Change in the measured relative intensity of BSA-based materials in the presence of ethanol, monitored with 8-Anilinonaphthalene-1-Sulfonic Acid (ANS) 1 μM, a reporter for protein unfolding and oligomerization; **C)** A similar experiment performed in the presence of Thioflavin T 1 μM, a reporter for fiber formation.

Next, we utilized two fluorescent probes to monitor the changes in the internal structure of BSA-based biomaterials in various concentrations of ethanol. First, we used 1-Anilinonaphthalene-8-Sulfonic Acid (ANS) (Figure 2B). ANS serves as a fluorescent probe for exposed, loosely unfolded structures and can directly quantify structural changes in the tertiary structure of BSA.^30^ ANS displays a weak fluorescence in aqueous environments, while becoming strongly fluorescent with a blue shift from ∼515 nm to ∼475 nm when embedded in structured hydrophobic pockets.^30^ We note here that ANS produces a strong signal upon binding partially unfolded but still structured regions of BSA, where the α-helices of BSA remain structured. This signal is expected to disappear upon complete denaturation, or when aggregation takes place, or when the hydrophobic pockets are no longer solvent accessible.^27,31^ Our measurements showed a fast increase in ANS fluorescence, which plateaus <5 min (Figure 2B). When we quantified the measured ANS intensity at the 30 min time point, we noticed an increase up to 40% ethanol, followed by a plateau up to 80% and a sharp decrease in the 80–99% range (Figure 2B bottom right). A similar behavior was reported for BSA solutions in ethanol, with an intensity increase in the 30–50% interval, followed by a plateau up to 80% and a sharp decrease in the 80–99% ethanol concentration range.^20^ We note here that in the case of BSA, ethanol and ANS compete for the same three hydrophobic pockets that are also used by this protein *in vivo* to transport fatty acids through the bloodstream.^19^ While the binding constant of ANS to BSA is significantly higher than that of ethanol (5.8 × 10^4^ mol^–1^ vs 1.9 × 10^2^ mol^–1^ ref. 19), the conditions where ethanol exceeds 40% will be impacted by competitive binding. Nevertheless, the increase in ANS fluorescence of BSA-based biomaterials with ethanol concentration is indicative of an increase in the nonpolar environment or hydrophobicity, which was previously associated with fast-occurring oligomerization.^25^ The decrease in ANS fluorescence after 60% ethanol can be an indication of amorphous aggregation but also of the replacement of the ANS molecules by ethanol inside BSA.

This self-association of BSA molecules in destabilizing conditions is a precursor for fibril formation, having in their core β-sheet-rich amyloid-like aggregates.^25^ These β-sheet-rich fibril formations can be monitored with a different dye, Thioflavin T (ThT).^22,25,32,33^ ThT displays a weak fluorescence in aqueous environments, while becoming strongly fluorescent with a red shift from ∼430 nm to ∼482 nm when attached to β-sheet-rich fibrils.^32^ Our results with the ThT dye support the SEM images and confirm an increase in fibrils up to 80% ethanol, followed by a slow decrease from 80% to 99% ethanol (Figure 2C). This decrease is probably associated with higher-order amorphous aggregates, as can also be seen in SEM images (Figure 2A right). Unlike the ANS measured signal, which increases immediately with the addition of ethanol, the ThT-generated signal showed a lag time and a plateau was reached after >20 min (Figure 2C bottom-left). This lag time was associated previously with a two-phase process, requiring first nucleation through the formation of partially unfolded oligomeric intermediates, followed by a growth phase of these intermediates into fibrils through a slower self-assembly process.^26^ Fitting our data in Figure 2C with a standard nucleation model, we find an apparent elongation rate of ∼0.2 min^–1^ (Figure S2 and Table S2). These experiments also confirm the previously reported sequence of structural rearrangement, where the conformational transition from α-helices to extended regions occurs more rapidly than the increase in aggregate size and the formation of β-sheet-rich, amyloid-like structures.^25^ Furthermore, the nonmonotonic behavior, and especially the structural changes seen when ethanol was increased from 80% to 99%, can be related to the dramatic decrease in the solubility of BSA under these conditions.^20^

Formation of fibrils is expected to provide increased structural rigidity. To measure the effect of ethanol on the mechanical properties of BSA-based biomaterials, we employed our custom force-clamp rheometry setup, developed specifically for measuring soft low-volume hydrogels^27,34^ (Figure 3A). Our instrument utilizes an analog feedback mechanism which continuously adjusts the position of the pulling-end of the tethered cylindrical biomaterial to match the desired stress set-point. To obtain stress–strain curves of BSA biomaterials under various solution conditions, we ramped up and down the stress to 4 kPa over 100 s. The slope of the stress vs strain curves during the linear part of the force-increase was used to evaluate the stiffness of the BSA-made biomaterials, reported as Young’s moduli, while the hysteresis between the two regimes (increase and decrease of applied stress) produced the energy dissipation for each condition (Figure 3B and C). When the biomaterial was moved from saline buffer into water, the BSA-based biomaterials became stiffer, and this stiffness further increased with the amount of ethanol to which the biomaterial was exposed (Figure 3B). The increase in stiffness was accompanied by a decrease in the energy dissipated (Figure 3C). This decrease in energy dissipation is indicative of fewer protein domains being able to unfold under load and refold back upon stress release.^35^ The reversibility of these mechanical changes upon rehydration and cycling was evaluated in detail, demonstrating partial to full recovery of stiffness and energy dissipation depending on ethanol concentration (Figure S3 and Table S3). Furthermore, there was a considerable swelling in water and ethanol 20%, followed by shrinking thereafter, indicative of the loss in solubility (Figure 3D). Hence we reason that structured fibril formation competes with amorphous aggregation (Figure 3E). Structured fibrils within BSA biomaterials represent ordered, β-sheet-rich assemblies of partially unfolded protein molecules that align intermolecularly to form elongated nanostructures akin to amyloid-like fibers. These fibrils emerge under conditions that promote controlled denaturation, such as moderate ethanol concentrations, where the solvent disrupts native α-helical domains (typically comprising ∼67% of the secondary structure^36^) and encourages hydrophobic interactions to drive organized β-sheet stacking. This ordered architecture imparts significant mechanical reinforcement, enhancing the rigidity and load-bearing capacity of BSA-based biomaterials, as reflected in elevated Young’s moduli during rheological testing. In contrast, amorphous aggregation involves the chaotic clumping of more extensively denatured BSA molecules into irregular, nonfibrillar clusters without defined secondary structure motifs. This pathway predominates at higher ethanol levels, where rapid and intense protein unfolding outpaces the kinetics of ordered assembly, leading to partial network formation interspersed with large disorganized aggregates. Such amorphous structures contribute to greater material plasticity, allowing for more pronounced energy dissipation through unfolding-refolding of flexible domains but at the expense of overall stiffness and uniformity. The competition between these elements is finely tuned by ethanol concentration, with optimal levels favoring fibril dominance for robust, stiff hydrogels, while excessive ethanol shifts toward amorphous control, potentially compromising the gel integrity. This dynamic also leads to volumetric changes, where initial swelling in lower ethanol arises from hydrated amorphous regions, transitioning to shrinkage as compact fibrils expel solvent and densify the network (Figure 3D). The observed competition between structured fibril formation and amorphous aggregation in BSA biomaterials under varying ethanol concentrations may be further influenced by hydrodynamic stress, with environmental modulation controlling amyloid polymorphism, suggesting a potential mechanism for tuning the mechanical properties of our biomaterials.^37^

**Figure 3:**
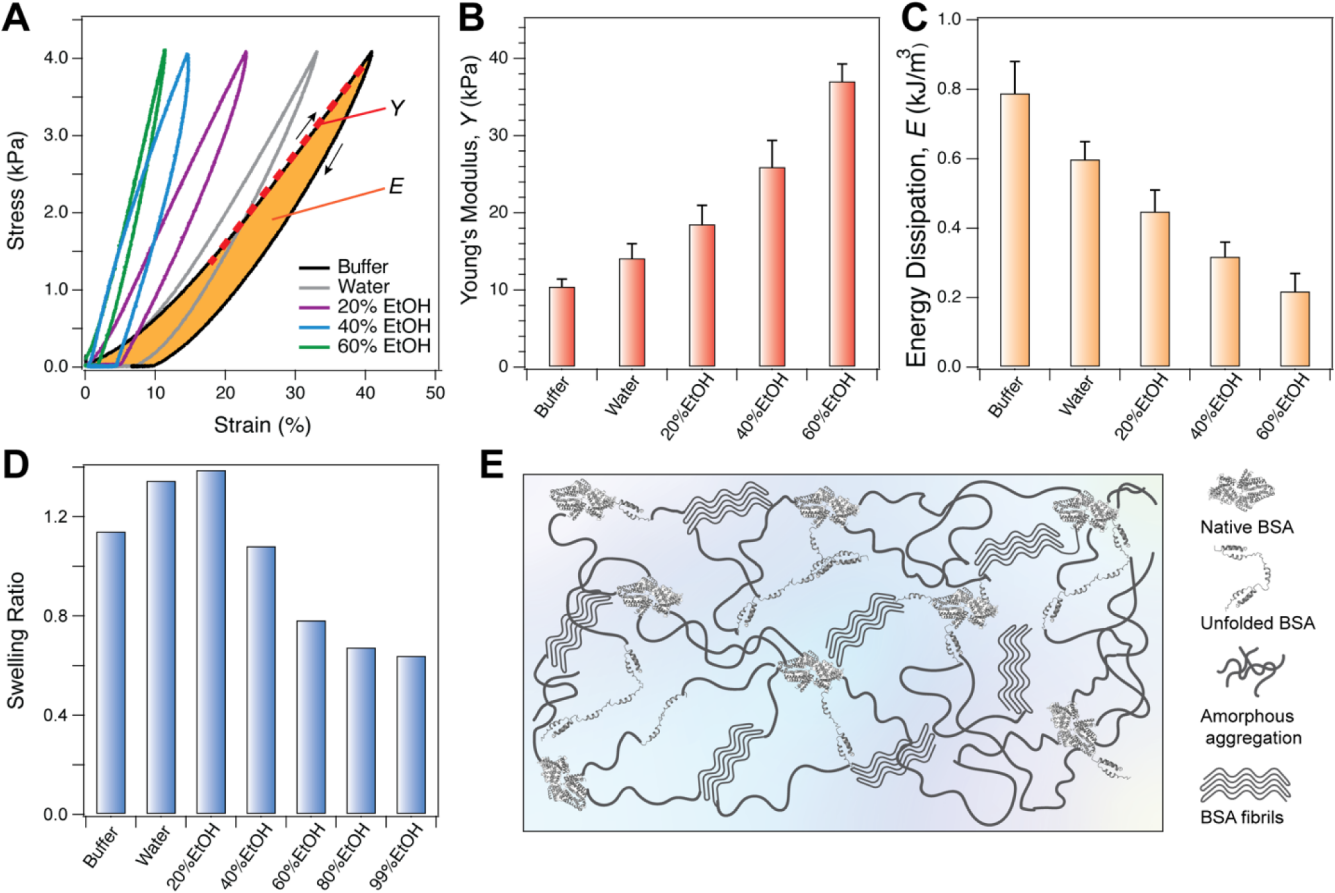
Mechanical characterization of BSA-based biomaterials. **A)** Stress–strain curves obtained under a controlled force-ramp protocol (40 Pa/s) in different buffer conditions. The slope of the red dotted line corresponds to the Young’s modulus (Y), while the orange hysteresis area gives the energy dissipation; **B)** Measured Young’s modulus, determined from the slope of the traces in panel A, as the load is increased (top parts of the traces), showing a monotonic increase with the added amount of ethanol; **C)** Energy dissipation, determined from the hysteresis area between the loading and unloading parts of the traces in panel A, showing a monotonic decrease with ethanol concentration. **D)** Swelling behavior of 2 mM BSA protein-based biomaterials in various solvent conditions after 30 min. **E)** Schematics of the inner structure of BSA-based biomaterials in the presence of ethanol, having folded and unfolded protein domains between areas of crystalline and amorphous structures.

### 3.2 Programmed Motion Induced by Reversible Stiffening

The ethanol-induced stiffening of BSA-based biomaterials offers a distinctive opportunity to utilize fibril formation for programming novel shapes. In previous reports, we showed that polyelectrolytes and divalent ions can stiffen these materials enough to enable shape programming and morphing.^11,13^ However, unlike the electrostatically driven stiffening mechanism, here, we are aiming at exploring fibril formation as a novel approach for shape programming. First, we characterized the capability of a BSA biomaterial, programmed in ethanol, to maintain its shape when it was taken out of a mold. Cylindrical biomaterials were programmed in a V-shape by immobilizing them between three 3D-printed pillars and exposing them to ethanol environments for 30 min (Figure 4A). The programmed biomaterials were then taken out of the mold and left in the same ethanol solution for an additional 30 min to see if they will retain their newly programmed shape. This capability is typically measured through the shape fixity parameter,^38,39^ which is defined as Rf=θtθp×100 , with *θ*_*t*_ being the temporarily fixed angle and *θ*_*p*_ the programmed angle. As seen for all of the conditions, except for 20% ethanol, the biomaterials retained their programmed shape when taken out of the mold in the same ethanol buffer (first 30 min Figure 4A bottom right) and had an excellent fixity of over 90% (Table S1). The loss of programmed shape in 20% ethanol buffer is in accord with previous findings that BSA in solution shows no change in the secondary structure in this solvent condition, and significant structural changes only start to occur above 30% ethanol concentration.^20^ Removal of ethanol was then done by first immersion in water, followed by saline buffer. The 40% and 60% ethanol maintained the highest bending angle upon removal of ethanol, while the 99% ethanol condition resulted in a loss of the programmed shape within the sampled time frame (1 h). While a good fixity in ethanol buffer is needed when removing the gel from the programming mold (Table S1), a large decrease is required when immersed in water/buffer to produce the transition from the programmed to the original shape. Hence, for shape recovery, the 99% ethanol exposure seems to be the most promising.

**Figure 4:**
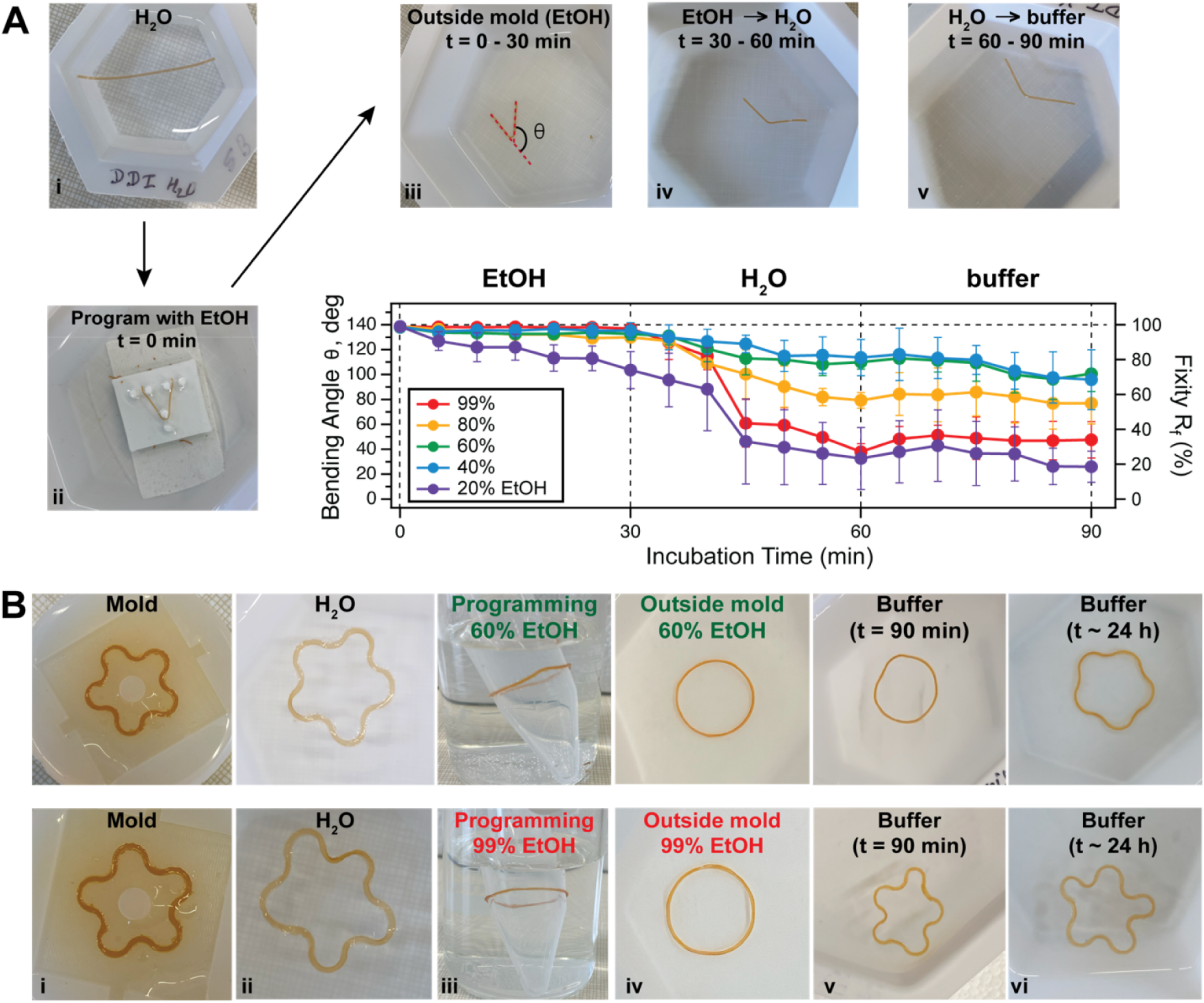
Shape-change induced by ethanol exchange in BSA-based biomaterials. **A)** Variation of the programmed angle from various ethanol conditions: **(i)** image of the BSA biomaterial synthesized in a cylindrical shape and washed for 30 min in water; **(ii)** the BSA biomaterial is then immobilized between three pedestals and programmed in a U-shape by immersion in a buffer containing ethanol; **(iii)** the biomaterial is removed from the mold and left to rest in the same ethanol solution for 30 min, then transferred in water for 30 more min **(iv)** and in Tris buffer **(v)**; (bottom-right): measured bending angle and fixity show that apart from the 20% ethanol condition, the BSA-based biomaterials maintain their programmed shape in the same ethanol buffer (first 30 min), followed by a decrease when moved to water/buffer, which is most pronounced to the highest ethanol condition; **B)** Picture showing a freshly synthesized BSA biomaterial in a flower-shaped mold **(i)**, and extruded in water **(ii)**, followed by programming in a ring shape inside ethanol using a 5 mL tube as a mold **(iii)**, then extruded in the same ethanol solution **(iv)**, where it maintains its shape, and then moved to water for 30 min followed by saline buffer for an additional 30 min **(v)** and 24 h **(vi)**; top row: programming done with 60% ethanol; bottom row: programming done with 99% ethanol.

Next, we tested the potential of using ethanol for shape-programming and shape-morphing through a more-complex ring-to-flower shape-morphing experiments that we previously introduced to measure solvent-induced transitions^13^ (Figure 4B and Figures S3–S5 and Movies S1–S2). In these experiments, we cross-linked BSA biomaterials in a flower shape (Figure 4B i and ii). We then immobilized them on a cylindrical form and immersed them in ethanol. The stiffening of BSA-based biomaterials in ethanol is expected to produce a programmed ring shape in this step (Figure 4B iii). Upon subsequent removal of the tube mold, the gels were left in the same ethanol buffer, where they maintained their ring shape (Figure 4B iv). They were then moved from ethanol into water and then saline buffer. A complete recovery will produce back the original flower shape, while irreversible programming will result in the biomaterial retaining the ring shape. As expected from our fixity experiments above, the best recovery of the original shape was seen with 99% ethanol within our sampled time (∼1 h) (Figure 4B v-bottom and Movie S2). Final shapes between a ring and a flower were seen for 40–80% ethanol conditions (Figure 4B v-top and Figure S4 and Movies S1). Hence, in these conditions the formation of fibers produces good programming, but poor recovery after 1 h, while the 99% condition produces both good programming and good recovery. Interestingly, when left overnight, the 40–80% too showed decent recovery, suggesting that the fiber formation, while stable, was not irreversible (Figure 4B vi and Figures S4–S5).

### 3.3 Solvation-Induced Motion Resembling Muscle-like Fast Responses

Apart from the interesting effect of ethanol on the structure of BSA-based biomaterials, another surprising observation was the gel behavior when immersed in ethanol and then back into water. Our initial observations were that the BSA-based biomaterials moved visibly faster and with stochastic fluctuations in this step. This nonequilibrium behavior was more clearly seen when one of the ends got attached to the wall of the hexagonal weighing boat used for solvent exchange, and the other end was still able to move freely (Figure 5A and Movie S3). The pulsating motion of the free end was described in terms of the fluctuation angle as a function of time. The average initial fluctuation rate of the BSA-based biomaterials varied between 0.5 and 1.5 pulsations/s when going from 40% to 99% ethanol into water. Unlike the slow motion driven by solvent exchange from our previous experiments (Figure 4), which take place over ∼30 min, these fluctuations were significantly faster, on the second timescale, and subsided after ∼30 s.

**Figure 5:**
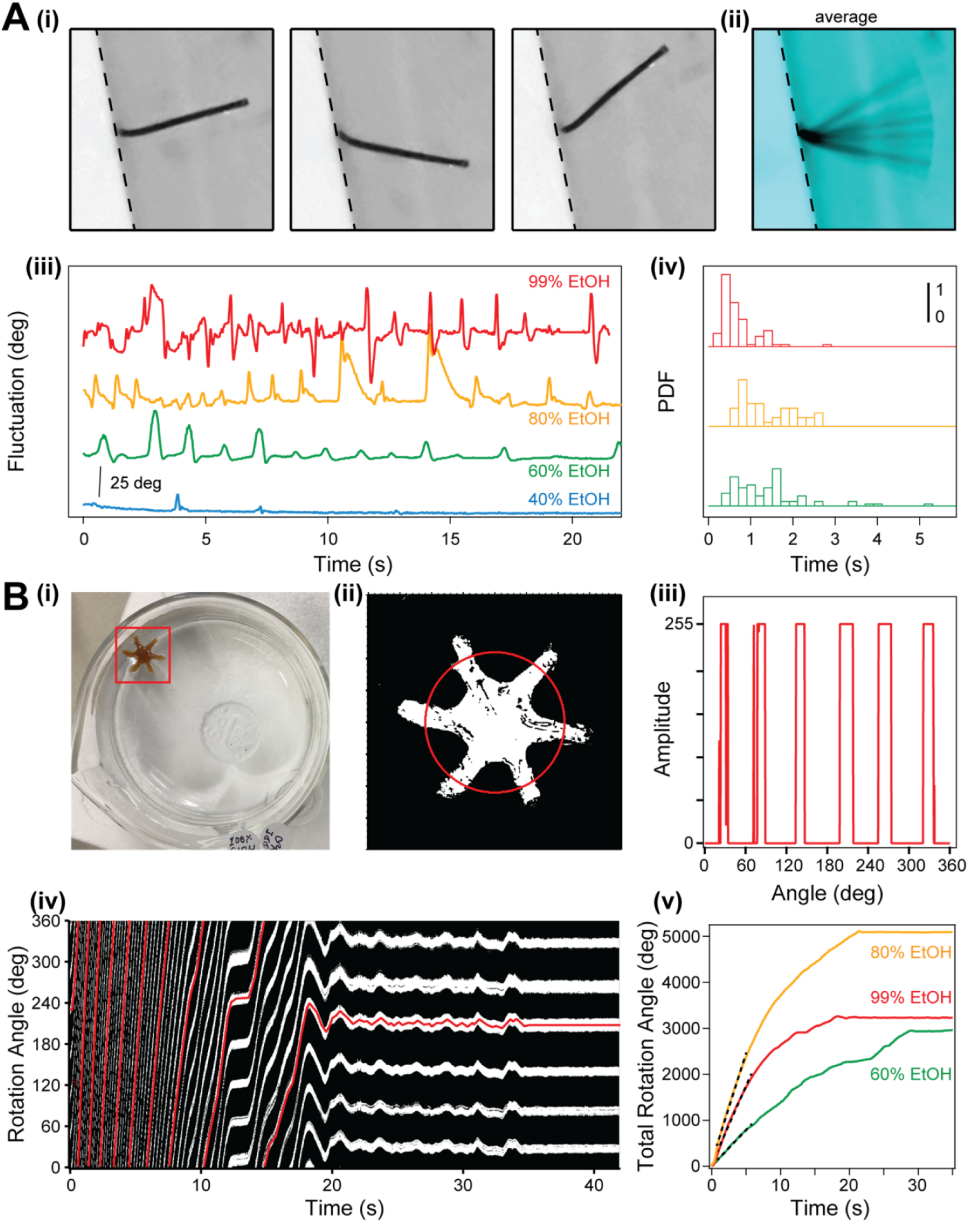
The effect of ethanol–water exchange on BSA-based biomaterials. **A)** Motion induced solvent exchange in cylindrical BSA-based biomaterials tethered at one end: **(i)** three snapshots of the same BSA-based biomaterial (gray-left) and **(ii)** the overall average (cyan); the dotted line marks the interface between the weigh boat and fluid; **(iii)** traces showing the measured fluctuations for BSA-based biomaterials immersed in water from different concentrations of ethanol; **(iv)** probability distribution function (PDF) of the measured fluctuations as a function of time for three ethanol concentrations; **B)** Motion-induced solvent exchange in a BSA-biomaterial having a six-arm propeller shape: **(i)** image of the propeller floating on top of water; the red square shows the ROI centered to the propeller, used to track and analyze the biomaterial; **(ii)** an image of the threshold calculated from the ROI and the circular line profile used to determine the orientation of each propeller (red); **(iii)** a representative line profile, where the presence of each propeller arm shows a sharp change in signal; **(iv)** change in the measured rotation angle as a function of time for a propeller immersed from ethanol into water, obtained by stacking the circular profiles from (iii); the red trace follows one of the propeller arm; **(v)** total average rotation angle of the BSA-propellers as a function of ethanol concentration and time; the dotted lines mark the initial change rate, which was 156 ± 1 deg·s^–1^ for 60% ethanol, 471 ± 2 deg·s^–1^ for 80% ethanol, and 340 ± 2 deg·s^–1^ for 99% ethanol; propellers immersed from ethanol concentrations of 40% or less did not float.

To fully take advantage of the potential of this solvent flux, we implemented a six-arm propeller design (Figure 5B, Movies S4–S6). BSA-based biomaterials were cast in this propeller shape and treated in a similar manner from saline buffer to ethanol. After resting for at least 30 min in a certain ethanol concentration (40–99%), the propellers were released on the water surface. Apart from the ones immersed in 40% ethanol, which sank to the bottom, the BSA propellers coming from the other conditions showed a surprising spinning motion (Figure 5B and Movies S4–S6). To quantify this rotation, we first implemented a tracking protocol, which applies a threshold to digitize the image, followed by a 2D Gauss fit to find the center of the propeller in each frame (see Section 2 for more detail). After cutting a region-of-interest (ROI) around the center of mass of the propeller, we then computed a circular profile (Figure 5B i to iii). Using these circular profiles, we then plotted the rotation topography as a function of time in a 2D plot (Figure 5B iv) and tracked the rotation of each of the six arms (one of the arms is shown in red). The average angular motion of the six arms then produced the total rotation angle (Figure 5B v). As can be seen from Figure 5B, the rotation speed was constant for ∼10 s for the 60% and 99% ethanol conditions and ∼15 s for the 80%, and the propeller stopped rotating after ∼30 s. Interestingly, the rotation speeds for 80% and 99% were very similar, while the highest rotation potential was seen at 80% ethanol, which rotated ∼50% longer. This potential seems to follow the fiber formation propensity, as measured with ThT and SEM experiments (Figure 2). Hence, we reason that this unexpected behavior comes from a solvent exchange, which can both generate a tidal-like flow and trigger rapid structural changes inside the protein-based biomaterial, while being influenced by the disassembly of fibrillar structures. This mechanism of inducing motion, driven by solvent exchange and the diffusion of solvent molecules out of the biomaterial network, could potentially be utilized in soft actuators and adaptive biomaterials.

The observed pulsating motion in cylindrical samples occurs on a second-scale time frame (1–3 s), and the rotational speeds of the BSA-based motor (156–471 deg·s^–1^) are inconsistent with the pure diffusive release of ethanol from the biomaterial. For regular diffusion in the cylindrical geometry used here, regular diffusion takes place with a characteristic time of ∼13 s (see SI Annex and Figure S6). This time scale implies that ethanol release would take tens to hundreds of seconds to equilibrate, yet the experimental motion ceases within ∼30 s. This discrepancy suggests that simple diffusion cannot account for the rapid actuation, and convective enhancement must be involved. Convection arises from the solutal Marangoni effect,^40,41^ where the ethanol gradient reduces surface tension, inducing tangential flow that thins the boundary layer and accelerates mass transfer. This convection effect shortens the characteristic time to milliseconds and the effective time scale to ∼30 s, consistent with observations. Control experiments done by moving the BSA biomaterials from ethanol into solutions with similar surface tension support this conclusion (Movies S7 and S8). In these control experiments, BSA biomaterials cast as both a cylinder and a propeller showed no motion when immersed from ethanol to 1% Triton X-100 solution. Triton X-100 1% decreases the surface tension of water from ∼72.8 mN/m to ∼30 mN/m,^42^ comparable to that of the ethanol solutions.^43^

## 4 Discussion and Conclusion

Here we show that protein-based biomaterials made from BSA can dynamically interact with ethanol solvents to produce programmed shapes. Instead of using a more common double-network approach, here we take advantage of the phase transition induced by the solvent to reshape the inner structure of the primary protein network. Ethanol both decreases the solubility of the protein and triggers its denaturation. Since BSA molecules are immobilized by the covalent network, the decrease in solubility can only manifest through solvent displacement and limited formation of aggregates. On the other hand, protein denaturation leads to a different phase transition that manifests itself through the formation of fibrils. The transition from disordered and partially unfolded proteins to ordered and insoluble fibrils is also marked by structural changes in the inner structure of BSA from the mostly α-helix structure of the native fold to cross-β sheets.^16^ Indeed, our topology and fluorescent dye measurements confirm that both these processes take place inside the biomaterial. We chose ANS to report on the exposure of hydrophobic regions of BSA in the presence of ethanol, which become exposed to the solvent during partial denaturation, oligomer assembly, or aggregation.^30^ This approach synergizes with ThT, which was employed as a reporter for the fibril formation of β-sheet-rich amyloid-like aggregates.^30^

ANS serves as an extrinsic hydrophobic probe to track amorphous aggregation and exposure of hydrophobic regions in BSA during ethanol-induced structural changes in the hydrogels. It binds to hydrophobic pockets or clusters, enhancing fluorescence upon burial in nonpolar environments, which signals partial unfolding, oligomerization, or aggregation. This complements ThT by distinguishing nonfibrillar (amorphous) aggregates, which dominate at high ethanol concentrations (80–99%) and allow reversible shape recovery. ANS helps elucidate how ethanol drives denaturation and aggregation outpacing fibrillation at high levels, linking these to the biomaterials’ reversible actuation and lack of cytotoxicity. Interestingly, above 80% ethanol content, the aggregation outpaces denaturation, resulting in a decrease in fibril formation above this concentration. While a similar behavior as a function of ethanol concentration was reported for BSA in solution, an explanation for this nonmonotonic process was never proposed.^20^

Both aggregation and fibril formation led to stiffening of the biomaterial, enabling programming into new shapes. Indeed, BSA biomaterials immersed in solutions with over 20% ethanol content were programmed in both V and cylindrical shapes from an original linear or flower profile, respectively. However, as fibril dissolution is a much slower process than the solvation of aggregates, immersing the programmed shapes back into water/saline buffer conditions only led to the recovery of the original shapes in the 99% ethanol condition within 60 min. Importantly, after ∼24 h, an improved recovery of the original profiles was seen also for the 40–80% ethanol experiments, suggesting that fibril formation is reversible.

Due to its folded structure transitioning to fibrils or aggregates, BSA can incorporate and retain large amounts of ethanol molecules, both specifically at fatty acid-binding sites and nonspecifically throughout its structure.^19,21^ This retention of ethanol inside the biomaterial led to a peculiar behavior when immersed back into water. Cylindrical BSA-based biomaterials exhibited fast, stochastic pulsating motion, which was more evident when one end was attached to the wall of the container, allowing for only the opposite end to fluctuate. We reason that these fast stochastic fluctuations are an effect of the combined diffusion and convection of ethanol. Localized bursts from fibril disassembly and heterogeneous fibril distribution probably lead to nonuniform ethanol release. Taking advantage of this behavior, we designed and implemented the first solvent-exchange-induced protein motor. This protein motor was designed to resemble an airplane propeller and showed fast rotative motion that lasted for up to 30 s.

The dynamic response of BSA-based biomaterials to ethanol–water solvent exchange opens new pathways for developing soft actuators and responsive biomaterials. The ability to reversibly program shapes using ethanol concentrations and recover original profiles in aqueous environments could be exploited in self-healing or shape-memory biomaterials, allowing for repeated programming cycles. The stochastic pulsating motion and the demonstrated protein motor resembling an airplane propeller highlight the potential for developing micro- and nanoscale actuators powered by solvent gradients and could be harnessed for microfluidic mixing and autonomous microscale robots. As solvent flux and hydrodynamic flow depend on the shape and size of the material, in cylindrical geometries, radial symmetry fosters stochastic pulsations, whereas the propeller’s asymmetric blades guide these flows into a directed torque. This shape dependence underscores the potential for geometric optimization to tailor actuation modes, from vibrational to rotational, in future bioactuators. Although the BSA-based motors reported here show actuation of the muscle-time scale of several seconds, the driving mechanism is fundamentally different: one is driven by ethanol flux and surface tension difference, while the other by ATP and myosin-actin power strokes. Further exploration of solvent combinations, protein modifications, and geometrical patterning represents a possible turning point of the field and could lead to a new class of sustainable, solvent-responsive bioactuators for applications ranging from smart textiles to biocompatible micromechanical systems.

## Supplementary Material





















## Notes

Movie S1. Shape-morphing induced by BSA fibril disassembly from 60% ethanol condition (MP4)
Movie S2. Shape-morphing induced by BSA fibril disassembly from 99% ethanol condition (MP4)
Movie S3. Solvent-exchange motion measured in an attached BSA cylinder when moved from ethanol to water (MP4)
Movies S4. Solvent-driven protein propeller motor for 60% ethanol initial conditions to water (MP4)
Movies S5. Solvent-driven protein propeller motor for 80% ethanol initial conditions to water (MP4)
Movies S6. Solvent-driven protein propeller motor for 99% ethanol initial conditions to water (MP4)
Movie S7. Comparison between the behavior of a cylindrical BSA biomaterial immersed from ethanol 80% to a solvent with similar surface tension and to water (MP4)
Movie S8. Comparison between the behavior of a BSA biomaterial with propeller shape immersed from ethanol 80% to a solvent with similar surface tension and to water (MP4)
Archived .stl file for the 3D mold used for the propeller experiment (ZIP)
Table S1. Fixity of BSA-based biomaterials in various concentrations of ethanol after 30 min. Table S2. Fitted values for fibril formation monitored by Thioflavin T (ThT) fluorescence. Table S3. Percentage increase or decrease of stiffness of BSA-based biomaterials under repeated solvent exchange. Figure S1. Size of BSA fibers. Figure S2. Influence of ethanol on BSA fibril formation monitored by ThT fluorescence. Figure S3. Mechanical stability of BSA-based biomaterials under repeated solvent exchange. Figure S4. Ring-shape programming and morphing to flower shape of BSA-based biomaterial using 40% and 80% ethanol. Figure S5. Analysis of shape-morphing experiments. SI Annex. Mathematical derivation of solvent flux driven actuation. Figure S6. Estimated ethanol flux as a function of time for a gel immersed in water from 80% ethanol (C ∼ 17 mol/L), through convective flow (Jconv) and diffusive flow (Jdiff) (PDF)
