# Supplementary material for: Protein Biomaterials with Muscle-like Water-Driven Actuation
Source: ACS Appl Mater Interfaces. 2026 Jan 3;18(1):1490–500. doi: 10.1021/acsami.5c19991 (PMC12781115; doi:10.1021/acsami.5c19991)
Supplement: Supplementary file 10 [file am5c19991_si_010.pdf]

SUPPORTING INFROMATION FOR

Protein Biomaterials with Muscle-Like Water-Driven Actuation

Sanam Bista, Ionel Popa

Department of Physics, University of Wisconsin-Milwaukee,
3135 N. Maryland Ave, Milwaukee, WI 53211, USA

Table S1 – Fixity of BSA-based biomaterials in various concentrations of ethanol, after 30 min

Ethanol concentration (%)	Fixity (%) $R_f = \theta_t / \theta_p \cdot 100$
20	74 ± 11
40	97 ± 8
60	95 ± 4
80	93 ± 1
99	98 ± 1

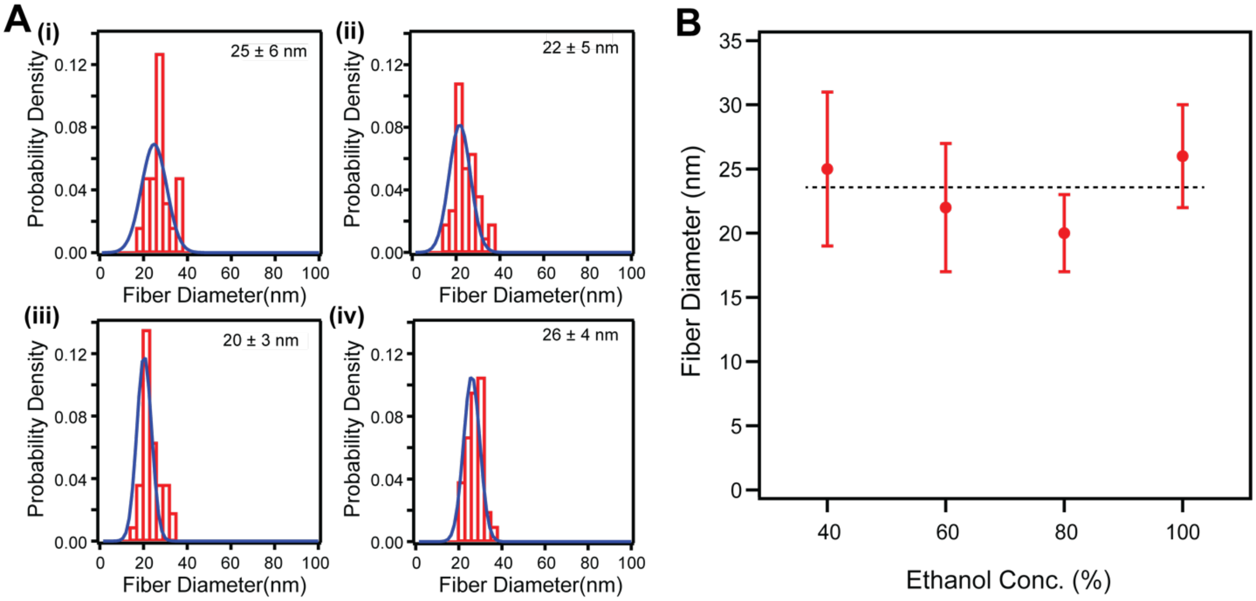

Figure S1. Size of BSA fibers. A) Nanofiber diameter obtained from SEM image of BSA-based biomaterial incubated in (i) 40% ethanol, (ii) 60% ethanol, (iii) 80% ethanol, and (iv) 99% ethanol; **B)** Distribution of nanofibers diameter of 2mM BSA-based biomaterial incubated in 40%, 60%, 80%, and 99% ethanol, respectively.

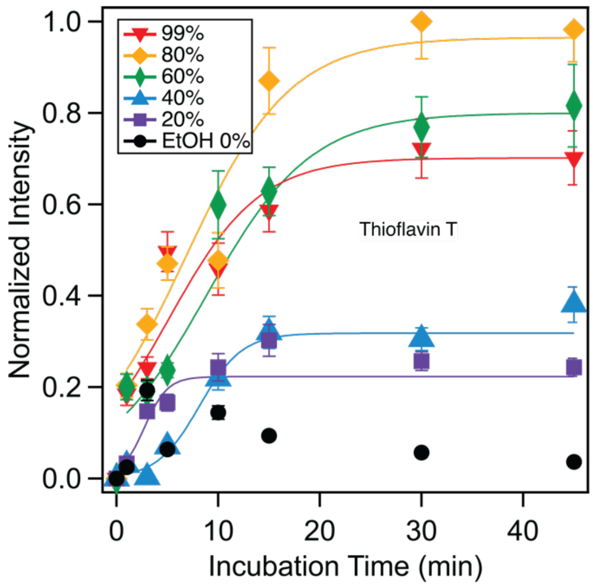

Figure S2. Influence of ethanol on BSA fibril formation, monitored by ThT fluorescence. ThT fluorescence data fitted using a standard sigmoidal model for amyloid fibrillation kinetics [1], which typically exhibit a lag phase followed by exponential growth and plateau, using $I(t) = \frac{A}{1+\exp[-k(t-t_{1/2})]}$, where $I(t)$ is the normalized intensity, k is the rate constant, and $t_{1/2}$ is the half-time, representing the time it takes for $I(t)$ to reach half of maximum intensity. The fitted values are shown in **Table S2.** Note that the error bar for 20% ethanol conditions is higher than for the others, and fit did not converge for no ethanol.

Table S2 – Fitted values for fibril formation monitored by Thioflavin T (ThT) fluorescence.

**% Ethanol**	***k*, min^{-1}**	**$t_{1/2}$, min**
20	0.9 ± 0.8	3 ± 1
40	0.5 ± 0.15	8 ± 1
60	0.19 ± 0.03	9 ± 1
80	0.19 ± 0.03	6.8 ± 0.8
99	0.22 ± 0.04	5.2 ± 0.8

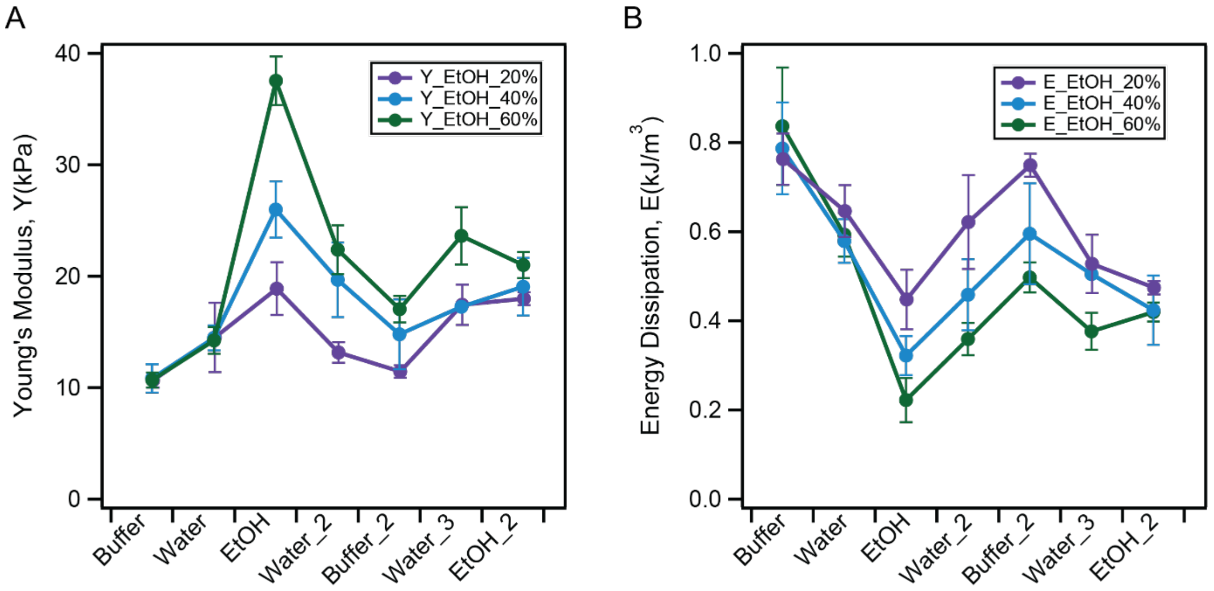

Figure S3. Mechanical stability of BSA-based biomaterials under repeated solvent exchange. a) Young's modulus of the same BSA-based materials measured sequentially after incubation in buffer, water, and ethanol (EtOH; 20%,40%, and 60% v/v). Measurements were repeated over multiple solvent exchange cycle to evaluate changes in stiffness. **b)** Energy dissipation of the materials during cyclic loading-unloading tests acquired after each solvent incubation step, illustrating variation in hysteresis behavior.

Table S3 – **Percentage increase or decrease of stiffness of BSA-based biomaterials under repeated solvent exchange.**

	Change Young's Modulus (%)		
	**20% Ethanol**	**40% Ethanol**	**60% Ethanol**
Water_2	-9.49%	31.96%	59.01%
Buffer_2	7.61%	36.57%	61.89%
Water_3	20.56%	19.23%	67.95%
EtOH_2	-4.86%	-26.68%	-44.56%

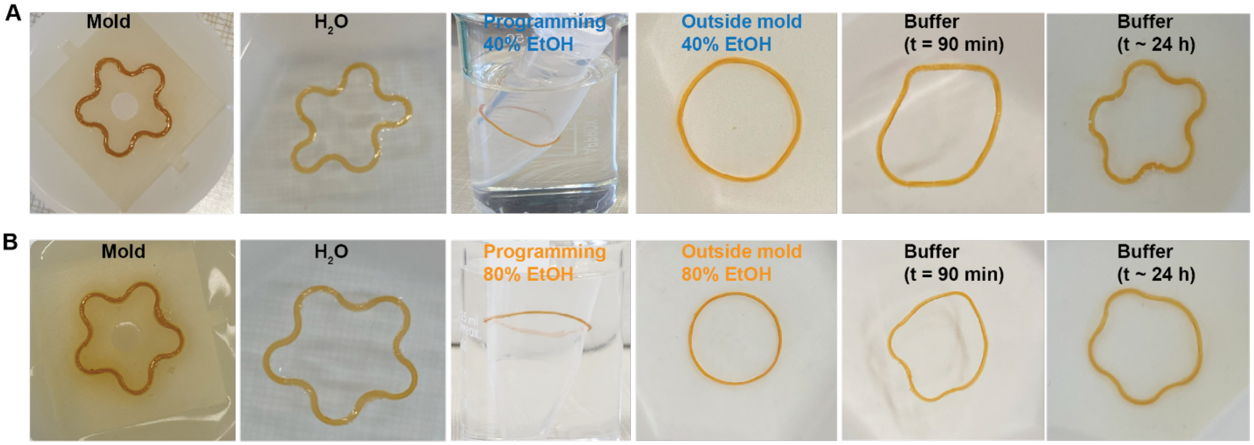

Figure S4. Ring-shape programing and morphing to flower-shape of BSA-bashed biomaterial using 40% and 80% ethanol. Pictures showing from left to right a freshly synthetized BSA-biomaterial in a flower-shaped mold, and extruded in DDI water, followed by programming in a ring shape by submerging into ethanol using a 5 mL tube as a mold, then extruded in the same ethanol solution, where it maintains its shape, and then moved to water and saline buffer for 30 min each, and the after ~24 hours; top row: programming done with 40% ethanol; bottom row: programming done with 80% ethanol.

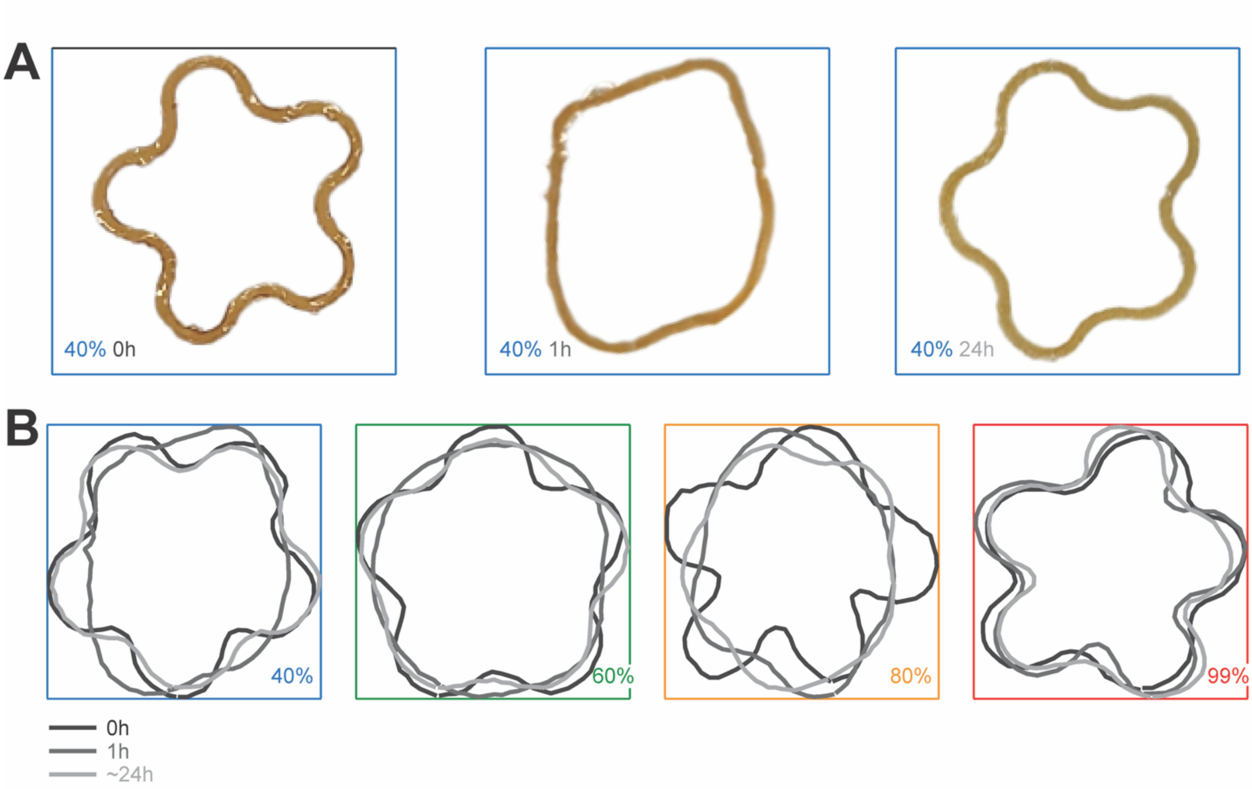

Figure S5. Analysis of shape morphing experiments. A) Picture showing a flower shape biomaterial before programming (0h), after programming into a ring in ethanol 40%, followed by 30 min in ethanol and 30 min in water and 30 min in saline buffer (a total of 1h in ethanol-free buffer) and after ~24 h in saline buffer (24h); B) Contour profiles for various ethanol-programming conditions, aligned to best overlap with the original shape.

SI Annex. Mathematical Derivation of Solvent Flux-Driven Actuation

The solvent (ethanol) diffuses radially from a cylindrical biomaterial of radius $a \approx 0.3\,mm$, with initial ethanol concentration $C_0 \approx 10 - 20\,mol/L$. Assuming Fick's second law in cylindrical coordinates, to model radial diffusion, we have the change in concentration as a function of time:

$$\frac{\partial C}{\partial t} = D\left(\frac{1}{r}\frac{\partial}{\partial r}\left(r\frac{\partial C}{\partial r}\right)\right) \tag{1}$$

where the diffusion coefficient of ethanol in water is $D \approx 1.24 \cdot 10^{-9} m^2/s$ [2], with the boundary conditions $C(r,0) = C_0$, $C(a,t) = 0$ and $\frac{\partial C}{\partial r}(0,t) = 0$, for the initial concentration (uniform concentration at $t = 0$), the concentration of ethanol at the surface (infinite sink at $r = a$) and the center boundary condition (no flux at $r = 0$), respectively.

Using separation of variables $C(r,t) = R(r)T(t)$, eq. **(1)** can be re-written [3]:

$$\frac{1}{T}\frac{dT}{dt} = D\frac{1}{rR}\frac{d}{dr}\left(r\frac{dR}{dr}\right) = -\lambda \tag{2}$$

where $-\lambda$ is the separation constant (negative for exponential decay in time). The time part then simply becomes $T(t) = T_0 e^{-\lambda t}$, and the radial part can be represented by:

$$r^2\frac{d^2R}{dr^2} + r\frac{dR}{dr} + \alpha^2 r^2 R = 0 \tag{3}$$

with $\alpha^2 = \lambda/D$. This is the Bessel's equation of order zero, and its general solution:

$$R(r) = AJ_0(\alpha r) \tag{4}$$

where J_0 is the Bassel function of the first kind, order zero. Given the infinite sink assumption, at $r = a$, we have $J_0(\alpha r) = 0$, which has the positive roots α_n, and corresponding eigenvalues $\lambda_n = D\alpha_n^2$. The general solution is a superposition:

$$C(r,t) = \sum_{n=1}^{\infty} A_n J_0(\alpha_n r)e^{-D\alpha_n^2 t} \tag{5}$$

At $t = 0$, eq. **(5)** becomes a Fourier-Bessel series $C_0 = \sum_{n=1}^{\infty} A_n J_0(\alpha_n r)$, with the coefficients A_n given by:

$$A_n = \frac{2}{a^2 J_1^2(\alpha_n a)}\int_0^a rC_0 J_0(\alpha_n r)dr = \frac{2C_0}{\alpha_n a J_1(\alpha_n a)} \tag{6}$$

using the orthogonality of Bessel functions. Thus, the concentration becomes:

$$C(r,t) = \sum_{n=1}^{\infty} \frac{2C_0}{\alpha_n a J_1(\alpha_n a)} J_0(\alpha_n r)e^{-D\alpha_n^2 t} \tag{7}$$

The radial flux at the surface ($r = a$) is:

$$J(t) = -D\left.\frac{\partial C}{\partial r}\right|_{r=a} = +\frac{2DC_0}{a}\sum_{n=1}^{\infty} e^{-D\alpha_n^2 t} \tag{8}$$

The expected flux decay, given the size of our cylindrical gel, is:

$$\tau_{diffusion} \approx \frac{1}{D\alpha_1^2} = \frac{a^2}{D(2.405)^2} \approx 13\ s \tag{9}$$

The observed motion (<3 s for tethered cylindrical gels) is faster than this characteristic decay time. Hence we reason that an addition convective flow must play a role. This flow can result from the surface tension gradient that forms at the material-water interface, and can be described using the Marangoni fluid transfer [4]. Assuming a surface tension gradient:

$$\gamma = \gamma_0 + C_0\frac{d\gamma}{dc} \tag{10}$$

the corresponding Marangoni stress is given by:

$$\sigma_M = \frac{d\gamma}{dC}\frac{\partial C}{\partial r} \tag{11}$$

with the rate of change of the surface tension being $\frac{d\gamma}{dC} \sim -2.5 \cdot 10^{-5}\ N\ m^2/mol$ for ethanol-water mixtures[5]. The expected flow velocity, assuming Stokes flow and a boundary layer thickness $h \approx \sqrt{Dt}$, is [6]:

$$u \approx \frac{\sigma_M h}{\mu} \approx \frac{d\gamma}{dC}\frac{C_0}{a}\frac{\sqrt{Dt}}{\mu} = \sqrt{\frac{D \cdot Ma}{t}} \tag{12}$$

where the viscosity is $\mu = 10^{-3} Pa \cdot s$, and the Maroni number [7] is $Ma = \frac{\Delta\gamma h}{\mu D} = -\frac{C_0 a \frac{d\gamma}{dc}}{\mu D} \approx 10^5$. Hence, the convection time is much shorter:

$$\tau_{convection} \approx \frac{a^2}{D \cdot Ma} \approx 0.007\ s \tag{9}$$

Figure S4 shows how for the convective flow (enhanced by Marangoni effects), the flux is increased due to a thinner effective boundary layer, compared to regular flow-induced mixing.

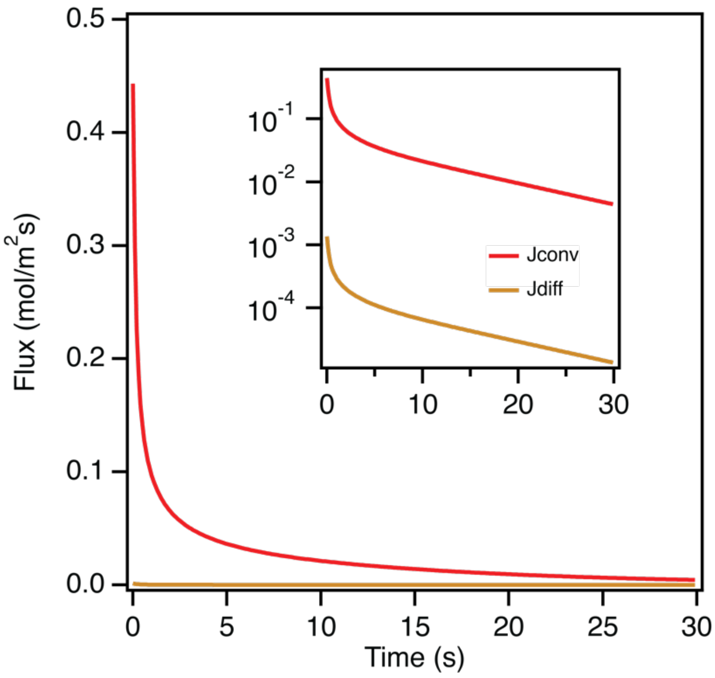

Figure S6. Estimated ethanol flux as a function of time for a gel immersed in water from 80 % ethanol (C ~ 17 mol/L), through convective flow (Jconv) and diffusive flow (Jdiff).

References

1. Nielsen, L.; Khurana, R.; Coats, A.; Frokjaer, S.; Brange, J.; Vyas, S.; Uversky, V. N.; Fink, A. L., Effect of environmental factors on the kinetics of insulin fibril formation: elucidation of the molecular mechanism. *Biochemistry* **2001,** *40* (20), 6036-46.
2. Pratt, K. C.; Wakeham, W. A., The mutual diffusion coefficient of ethanol–water mixtures: determination by a rapid, new method. *Proceedings of the Royal Society of London. A. Mathematical and Physical Sciences* **1974,** *336* (1606), 393-406.
3. Crank, J., *The Mathematics of Diffusion (2nd ed.)*. Oxford University Press, Oxford 1975.
4. Fanton, X.; Cazabat, A. M., Spreading and Instabilities Induced by a Solutal Marangoni Effect. *Langmuir* **1998,** *14* (9), 2554-2561.
5. Vazquez, G.; Alvarez, E.; Navaza, J. M., Surface Tension of Alcohol Water + Water from 20 to 50 .degree.C. *Journal of Chemical & Engineering Data* **1995,** *40* (3), 611-614.
6. Levich, V. G., *Physicochemical Hydrodynamics*. Prentice-Hall: 1962.
7. Kim, H.; Muller, K.; Shardt, O.; Afkhami, S.; Stone, H. A., Solutal Marangoni flows of miscible liquids drive transport without surface contamination. *Nature Physics* **2017,** *13* (11), 1105-1110.
